# Goal Identification Control Using an Information Entropy-Based Goal Uncertainty Metric †

**DOI:** 10.3390/e21030299

**Published:** 2019-03-20

**Authors:** Kai Xu, Quanjun Yin

**Affiliations:** College of System Engineering, National University of Defense Technology, Changsha 410000, China

**Keywords:** goal uncertainty, goal recognition, goal identification control, information entropy

## Abstract

Recent research has found situations where the identification of agent goals could be purposefully controlled, either by changing the underlying environment to make it easier, or exploiting it during agent planning to delay the opponent’s goal recognition. The paper tries to answer the following questions: what kinds of actions contain less information and more uncertainty about the agent’s real goal, and how to describe this uncertainty; what is the best way to control the process of goal identification. Our contribution is the introduction of a new measure we call *relative goal uncertainty (rgu)* with which we assess the goal-related information that each action contains. The *rgu* is a relative value associated with each action and represents the goal uncertainty quantified by information entropy after the action is taken compared to other executable ones in each state. After that, we show how goal vagueness could be controlled either for one side or for both confronting sides, and formulate this goal identification control problem as a mixed-integer programming problem. Empirical evaluation shows the effectiveness of the proposed solution in controlling goal identification process.

## 1. Introduction

Goal recognition—the ability to recognize the plans and goals of other agents—enables humans, AI agents, or command and control systems to reason about what the others are doing, why they are doing it, and what they will do next [1]. Until now, goal recognition system has worked well in many applications such as human–robot interaction [2], intelligent tutoring [3], system-intrusion detection [4] and security applications [5].

**Figure 1:**
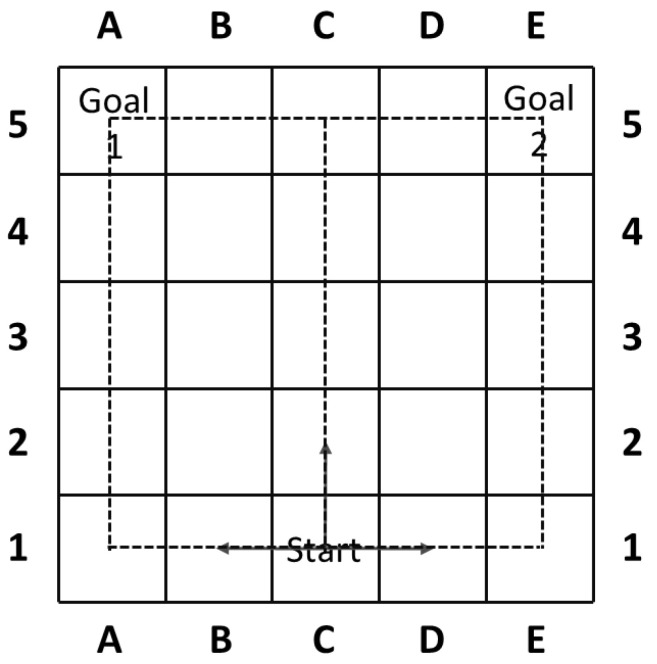
An example where goal identification process could be controlled, where C1 is the Start, and A5 and E5 are exit points denoted as Goal 1 and Goal 2, respectively. In this example, path ‘C1-C2-C3-C4-C5-B5-A5’ contains the largest goal uncertainty, while ‘C1-B1-A1-A2-A3-A4-A5’ the least.

Though this technique has been successfully applied to many application domains, new problems arise when goal recognition encounters a goal uncertainty discovered in certain environmental settings. Figure 1 offers a simple example that is first mentioned in [6] and will help to clarify the concepts of our work, which is extended from our original [7].

The model consists of a simple room (or airport) with a single entry point, marked as ‘Start’ and two possible exit points (boarding gates), marked as ‘Goal 1’ (domestic flights) and ‘Goal 2’ (international flights). An agent can move vertically or horizontally from ‘Start’ to one of the goals. Notice that in this model, the goal of the agent becomes clear once turning left or right, while moving vertically would impose goal uncertainty on goal recognizers and postpone the goal identification. Therefore, in the worst case an optimal agent can move up 4 steps before it is obliged to turn towards its goal.

The goal uncertainty showed in the above example could be viewed as an inherent property of one particular goal recognition task, and pose a new challenge to effective goal reasoning. The problem finds itself relevant in many of the similar applications of goal recognition tasks where goal uncertainty exists. Also, it should be noted that apart from the friendly situation in Figure 1 and works in [6], the goal uncertainty could further be used as a potential backdoor for the adversary to delay goal identification.

Therefore, this paper focuses on effective control of the goal identification process. As a first stage of our exploration, we assume agents are optimal and that the actions of the agent are fully observable and deterministic. To achieve our objective, we introduce a new concept called *relative goal uncertainty (rgu)*, with which we assess the goal-related information that each action contains. The *rgu* is a relative value associated with each action that agent would take and represents the goal uncertainty quantified by information entropy after the action is taken compared to other executable ones in each state.

The *rgu* value associated with each action helps in assessing the uncertainty that exists in goal recognition task, and our goal is to provide methods for agents to control it. Towards this end, we define two optimization models based on mixed-integer programming, one of which reduces the goal uncertainty through limiting the set of available actions an agent can perform, the other one delays goal identification through balancing the reward and goal uncertainty during mission planning.

Goal identification control, while relevant to goal recognition [1] or goal reasoning, is a different task. While goal recognition aims at discovering the goals of an agent according to observations, goal identification control rather offers an offline and online combined solution for assessing and controlling the goal uncertainty to assure the goal of any optimal agent in the system is recognized. Also, different from goal recognition design problem [6,8,9] which purely focuses on facilitating goal recognition offline, goal identification control provides a more compact solution for agents to control the goal uncertainty and allows this additional information to be incorporated into ongoing missions.

Finally, considering the situation where both confronting sides are trying to control the goal identification process (i.e., both sides are behaving in a strategic game-theoretic manner), the paper then proposes the Shortest-Path Largest-Uncertainty Network Interdiction model (SPLUNI), which could be viewed as a bilevel mixed-integer program.

The SPLUNI model is transformed into its dual form using KKT conditions and computed using mixed-integer programming method.

The paper is organized as follows. We start by providing the necessary background on probabilistic goal recognition. We continue by introducing the formal model representing the goal identification control problem, and the *rgu* value. The following sections present the methods we developed for calculating *rgu* and controlling the goal uncertainty of a given goal identification control problem. We conclude with an empirical evaluation, a discussion of related work, and a conclusion.

## 2. Background and Related Work

### 2.1. Probabilistic Goal Recognition

The goal recognition problem has been formulated and addressed in many ways, as a graph covering problem upon a plan graph [10], a parsing problem over grammar [11,12,13,14], a deductive and probabilistic inference task over a static or Dynamic Bayesian Network [15,16,17,18,19,20] and an inverse planning problem over planning models [21,22,23,24,25].

Among the approaches viewing the goal or plan recognition as an uncertainty problem, two formulations appear and solve the problem from different perspectives. One focuses on constructing a suitable library of plans or policies [16,17], while the other one takes the domain theory as an input and use planning algorithms to generate problem solutions [21,23]. Before reviewing these two formulations, we would first give the definition of probabilistic goal recognition (or plan recognition) as follows:

**Definition** **1.** *A probabilistic goal recognition problem or theory is a tuple T=〈D,G,O,Prob〉 where D is the problem domain, G is the set of possible goals G, $O=o_{1},\ldots ,o_{m}$ is an observation sequence (which may or may not be adjacent) with each $o_{i}$ being an action, and Prob represents the prior probabilities over G.*

And generally, the posterior goal distribution P(G|O) will be computable from the Bayes Rule as:

(1) P(G|O)=αP(O|G)P(G),

where α is a normalizing constant, and P(G) is Prob(G).

The uncertainty problem is first addressed by the work [16] which phrases the plan recognition problem as the inference problem in a Bayesian network representing the process of executing the actor’s plan. It is followed by more work considering dynamic models for performing plan recognition online [12,13,26,27]. While this offers a coherent way of modelling and dealing with various sources of uncertainty in the plan execution model, the computational complexity and scalability of inference is the main issue, especially for dynamic models. In [17], Bui et al. proposed a framework for online probabilistic plan recognition based on the Abstract Hidden Markov Models (AHMM), which is a stochastic model for representing the execution of a hierarchy of contingent plans (termed *policies*). Scalability in policy recognition in the AHMM, and the inference upon its transformed Dynamic Bayesian Network (DBN), is achieved by using an approximate inference scheme known as the Rao-Blackwellized Particle Filter (RBPF) [28]. Generally, in a particle system, the posterior distribution is empirically represented using a weighted sum of $N_{p}$ samples $g_{i}$ drawn independent identically distributed from the proposal distribution [29]:

(2) $$p\left(g|O\right)\approx \sum_{i=1}^{N_{p}}W_{i}\delta \left(g-g_{i}\right)$$

where the importance weights $W_{i}$ should be updated recursively.

Within this formulation, following research continues to improve the model’s expressiveness and computational attractiveness, as in [18,20,30,31,32,33,34,35,36]. Notably, research in [33,36] proposes a CSSMs model which allow simple system model definition in the form of planning problem and can reason in large state spaces by using approximate inference. Also, recently machine learning methods including reinforcement learning [35], deep learning [3,37] and inverse reinforcement learning [38,39] have already been successfully applied in learning the agents’ decision models for goal recognition tasks. These efforts once again extend the usability of policy-based probabilistic goal recognition methods by constructing agents’ behavior models from real data.

Recently, the work [21,22] shows that plan recognition can be formulated and solved using off-the-shelf planners, follows by work considering suboptimality [23] and partial observability [24]. Not working over libraries but over the domain theory where a set G of possible goals is given, this generative approach solves the probabilistic goal recognition efficiently, provided that the probability of goals is defined in terms of the ***cost difference*** of achieving the goal under two conditions: complying with the observations, and not complying with them.

(3) $${\mathrm{costdif}}_{RG}\left(s,g,O\right)=\mathrm{optc}\left(s,O,g\right)-\mathrm{optc}⌝\left(s,O,g\right)$$

By comparing cost differences across goals, a probability distribution is generated that conforms to the intuition: the lower the cost difference, the higher the probability. Concretely, they propose the assumption of a Boltzmann distribution and take P(O|G)=sigmoid(β(optc⌝(s,G,O)−optc(s,G,O))), thus arriving at the following posterior:

(4) $$P\left(G|O\right)=\alpha \frac{e^{-\beta X}}{1+e^{-\beta X}},$$

where $X={\mathrm{costdif}}_{RG}\left(s,G,O\right)$, α is a normalizing constant across all goals, and β a positive constant which captures a ‘soft rationality’ assumption.

Several works followed this approach [25,40,41,42,43,44] by using various automated planning techniques to analyze and solve goal or plan recognition problems. Its basic ideas have also been applied in new problems such as Goal Recognition Design [6], Deceptive Path-Planning [45], etc. The advantages of the latter formulation are two-fold: one is that by using plenty of off-the-shelf model-based planners the approach scales up well, handling domains with hundreds of actions and fluents quite efficiently; the other one lies in the fact that the model-based method has no concerns about the recognition of joint plans for achieving goal conjunctions. Joint plans come naturally in the generative approach from conjunctive goals, but harder to handle in library-based methods.

According to the relationships between the observer and the observed agent, the goal or plan recognition could be further divided into *keyhole*, *intended* and *adversarial* types [4,46,47,48]. In keyhole plan recognition, the observed agent is indifferent to the fact that its plans are being observed and interpreted. The presence of a recognizer who is watching the activity of the planning agent does not affect the way he plans and acts [26,49]. Intended recognition arises, for example, in cooperative problem-solving and in understanding indirect speech acts. In these cases, recognizing the intentions of the agent allows us to help or respond appropriately [46]. In adversarial recognition, the observed agent is hostile to the observation of his actions and attempts to thwart the recognition [50,51].

### 2.2. Goal Recognition Design

**Figure 2:**
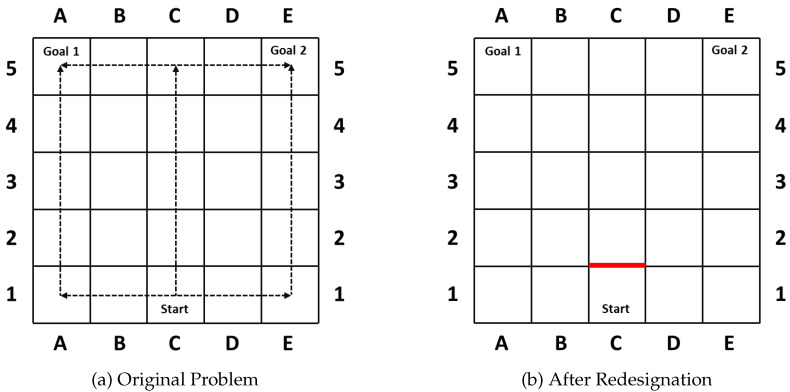
The motivating example of the GRD problem, where the *wcd* value is 4 and the blockade of the action moving the agent from C1 to C2 successfully reduces the *wcd* from 4 to 0.

Goal recognition design was first introduced in [6] to reduce goal uncertainty and advance the correct recognition through redesigning the underlying environment. It could be seen as an inverse problem to deceptive path-planning. Standing on the side of the observer, the GRD problem tries to reduce goal uncertainty and advance the correct recognition through redesigning the domain layout. To do so, they introduce a concept named *worst-case distinctiveness (wcd)*, measuring the maximal length of a prefix of a plan an agent may take within a domain before its real goal has been revealed. As in their first case [6] shown in Figure 2, the goal of the agent becomes clear once turning left or right, while it maintains the longest ambiguity if the agent moves straight up 4 steps (wcd=4) before it is obliged to turn towards its real goal. Thus, the blockade of the action moving the agent from C1 to C2 (Figure 2b) successfully reduces the *wcd* from 4 to 0, and prohibits the agent from hiding key information.

Since then, lots of research has been carried out. The work described in [6,52] extends the previous work by accounting for agents that behave non-optimally and non-observably. The work in [53] further allows the outcomes of agents’ actions to be non-deterministic, and proposes a *Stochastic GRD* problem. Apart from the relaxation of assumptions, researchers try to solve the GRD from different aspects, or use newly established metrics. Answer Set Programming [8] was first used to address the same problem, resulting in higher scalability and efficiency. Mirsky et al. [54] extend GRD to the Plan Recognition Design (PRD), which is the task of designing a domain using plan libraries to facilitate fast identification of an agent’s plan. Noticing the inconsistency between the original *wcd* (*worst-case distinctiveness*) and Stochastic GRD model, a new metric, namely *all-goals wcd ($wcd_{ag}$)*, is proposed by [9]. Also, based on new assumption of the availability of prior information about the agent’s true goal, they further propose a *expected-case distinctiveness (ecd)* metric that weighs the possible goals based on their likelihoods. Mirsky et al. [55] further study the goal and PRD for plan libraries.

The general GRD problem [6] is represented as a tuple P=〈D,G〉, where $D=\langle S,s_{0},A,f,C\rangle$ captures the domain information and *G* is a set of possible goal states of the agent. Except that all actions have the same cost of 1, the elements in the tuple *D* are as the definitions in classical planning where *S* is a finite and discrete state space; $s_{0}$ is the start state of the agent; *A* is the set of actions; f:S×A→S is a deterministic state transition function; and *G* is a set of goal states. The *wcd* of problem *P* is the length of a longest sequence of actions $\pi =\langle a_{1},\ldots ,a_{k}\rangle$ that is the prefix in cost-minimal plans $\pi_{g_{1}}^{★}$ and $\pi_{g_{2}}^{★}$ to distinct goals $g_{1},g_{2}\in G$. GRD’s objective is to find a subset of actions ${\hat{A}}^{★}\subset A$ such that if they are removed from the set of actions *A*, then the wcd of the resulting problem is minimized. As summarized in [53], the GRD task could be formulated into an optimization problem which is subject to the constraint that the cost of cost-minimal plans to achieve each goal g∈G is the same before and after removing the subset of actions:

(5) $$\hat{A}=\underset{\hat{A}\subset A}{\arg\min}wcd\left(\hat{P}\right)$$

$$\mathrm{subject}\mathrm{to}\;C\left(\pi_{g}^{★}\right)=C\left({\hat{\pi }}_{g}^{★}\right)\;\forall g\in G$$

where $\hat{P}=\langle \hat{D},G\rangle$ is the problem with the resulting domain $\hat{D}=\langle S,s_{0},A\backslash \hat{A},f,C\rangle$ after removing actions $\hat{A}$, $\pi_{g}^{★}$ is a cost-minimal plan to achieve goal *g* in the original problem *P*, and ${\hat{\pi }}_{g}^{★}$ is a cost-minimal plan to achieve goal *g* in $\hat{P}$.

## 3. Probabilistic Goal Recognition

**Figure 3:**
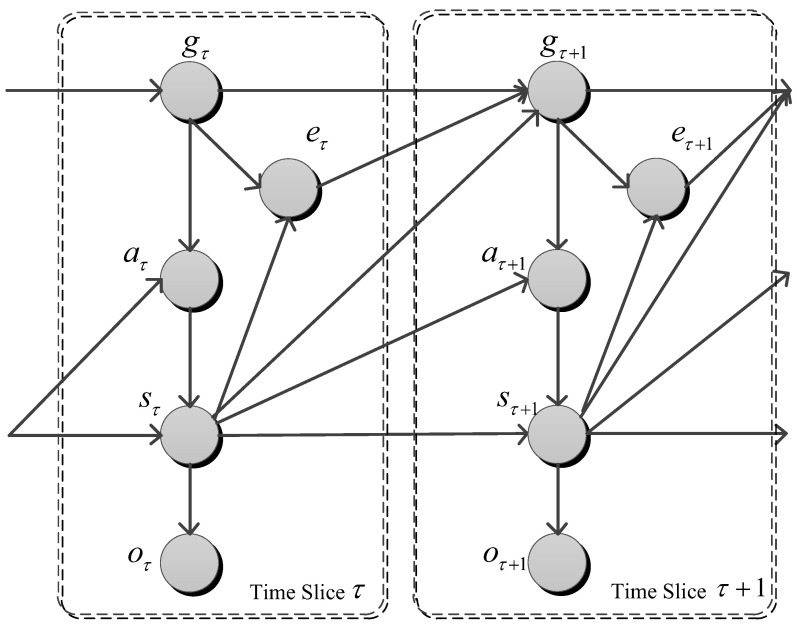
The DBN structure depicting causalities within two time slices of the model.

The authors in [20] shows a simple yet effective probabilistic goal recognizer based on Markov Decision Process (MDP). The probabilistic goal recognition model is defined by a tuple $D=\langle S,s_{0},A,f,G,e,O\rangle$, where *S* is a finite and discrete state space; $s_{0}$ is the start state of the agent; *A* is the set of actions; f:S×A→S is a deterministic state transition function; *G* is a set of goal states; *e* is the goal termination variable and *O* is a non-empty observation set. Essentially, the model is a DBN, in which all causalities could be depicted. We introduce a full DBN structure depicting two time slices is presented in Figure 3.

The behaviors of system evolution are described using functions or parameters.

- state transition function T:S×A×S→[0,1] is $P_{s_{\tau }}=p\left(s_{\tau }|s_{\tau -1},a_{\tau }\right)$,
- observation function S×O→[0,1] is $P_{o_{\tau }}=p\left(o_{\tau }|s_{\tau }\right)$,
- agent action policy $P_{a_{\tau }}=p\left(a_{\tau }|s_{\tau -1},g_{\tau }\right)$,
- goal transition probability $P_{g_{\tau }}=p\left(g_{\tau }|e_{\tau -1},g_{\tau -1}\right)$,
- goal termination probability $P_{e_{\tau }}=p\left(e_{\tau }|g_{\tau },s_{\tau }\right)$.

Recognizing the evader’s goal is an inference problem trying to find the real goal behind agent actions based on observations online. In essence, the task is to compute the posterior distribution $P(g_{\tau }|o_{\tau })$ of goal $g_{\tau }$ given observation $o_{\tau }$. This could be achieved either by accurate inference or by approximate methods. Accurate inference, however, is not scalable when state space of the domain problem becomes large, nor can it tackle partially missing or noisy data. Widely applied in sequential state estimation, particle filter is a kind of approximate inference methods designed to handle non-Gaussian and nonlinear problems [29]. As for the problem with a large-scale state space, methods such as [28,56] are preferred. In this work, we would like to simply show how the probabilistic goal recognition works, and thus the basic particle filter would be enough. In this work, the MDP or agent action model is assumed to be known by both the evader and the indicator, except for the current goal $g_{\tau }$ of the evader. Instead, the set of possible goals is given along with the priors P(G). Similar assumptions also exist in [24] in which the posterior goal probabilities P(G|O) is obtained from Bayes Rule

(6) P(G|O)=αP(O|G)P(G)

where α is a normalizing constant. In particle filter however, a posterior distribution is empirically represented using a weighted sum of $N_{p}$ samples [29] drawn from the proposal distribution:

(7) $$p\left(g_{\tau }|o_{\tau }\right)\approx \sum_{i=1}^{N_{p}}W_{\tau }^{\left(i\right)}\delta \left(g_{\tau }-g_{\tau }^{\left(i\right)}\right)$$

where $g_{\tau }^{\left(i\right)}$ are assumed to be *i.i.d* drawn from $q(g_{\tau }|o_{i})$. The importance weights $W_{\tau }^{\left(i\right)}$ should be updated recursively

(8) $$W_{\tau }^{\left(i\right)}\approx W_{\tau -1}^{\left(i\right)}\frac{p\left(o_{\tau }|g_{\tau }^{\left(i\right)}\right)p\left(g_{\tau }^{\left(i\right)}|g_{\tau -1}^{\left(i\right)}\right)}{q(g_{\tau }^{\left(i\right)}|g_{0:\tau -1}^{\left(i\right)},o_{\tau })}$$

As we use simplest sampling, the $q(g_{\tau }^{\left(i\right)}|g_{0:\tau -1}^{\left(i\right)},o_{\tau })$ is set to be $p(g_{\tau }^{\left(i\right)}|g_{\tau -1}^{\left(i\right)})$, which could be computed directly using the agent action model:

(9) $$p\left(g_{\tau }^{\left(i\right)}|g_{\tau -1}^{\left(i\right)}\right)\;=\;\int_{a_{\tau -1}^{\left(i\right)}}\int_{s_{\tau -1}^{\left(i\right)}}\int_{e_{\tau -1}^{\left(i\right)}}p_{g_{\tau }^{\left(i\right)}}p_{e_{\tau -1}^{\left(i\right)}}p_{s_{\tau -1}^{\left(i\right)}}p_{a_{\tau -1}^{\left(i\right)}}$$

Thus, the $g_{\tau }$ in Equation (7) would be sampled from $p(g_{\tau }^{\left(i\right)}|g_{\tau -1}^{\left(i\right)})$. As the observation $o_{\tau }$ only depends on $s_{\tau }$, the importance weights $W_{\tau }^{\left(i\right)}$ can be updated by

(10) $$W_{\tau }^{\left(i\right)}=W_{\tau -1}^{\left(i\right)}\cdot p\left(o_{\tau }|s_{\tau }^{\left(i\right)}\right).$$

In this work, a sequential importance sampling (SIS) filter with resampling is used in evader’s goal inference. Notably, in the resampling process, we apply the estimated *effective sample size*, ${\hat{N}}_{eff}$, according to

(11) $${\hat{N}}_{eff}=\frac{1}{\sum_{i=1}^{N_{p}}{\left({\tilde{W}}_{t}^{\left(i\right)}\right)}^{2}}$$

where ${\tilde{W}}_{t}^{\left(i\right)}$ is the normalized weight. The resampling process returns if ${\hat{N}}_{eff}>N_{T}$, where $N_{T}$ is the pre-defined threshold which is set to $N_{p}/3$, otherwise generates a new particle set by resampling with replacement $N_{p}$ times from the previous set with probabilities ${\tilde{W}}_{t}^{\left(i\right)}$, and then reset the weights to $1/N_{p}$.

## 4. Relative Goal Uncertainty

Given the set of possible goal states *G* and the current observation $o_{\tau }$, the probability distribution over possible goals $P(G|o_{\tau })$ actually reflects the goal uncertainty the agent will encounter after selecting actions $A(s_{\tau })$ and reaches the next state $o_{\tau }$ (assuming fully observable and deterministic) according to *f*. This enlightens us to use the goal inference information one step ahead to measure the goal uncertainty associated with available actions at each state, specifically the adjacent edges directing out of each node in the road network example.

Defined over each possible action $a_{i}\in A\left(s\right)$ at state *s*, the metric *rgu* is a relative value describing the overall uncertainty performance of state *s* before and after the action *a*’s interdiction. Intuitively, this could be done by summing up the information entropy of the pre-computed, next-step goal probability which are reasoned about in the goal recognition problem $D=\langle S,s_{i},A,f,G,e,O=\left\{s_{i},s_{j}\right\}\rangle$, where $s_{i}\in S$ and $s_{j}\in FS\left(s_{i}\right)$, the set of states that could arrive at according to $f(s_{i})$.

**Definition** **2.** **(Entropy-based *rgu*)** *The* Entropy-based *rgu ($rgu_{E}$) over a goal identification control problem is defined as:*

(12) $$\begin{array}{c} rgu_{E}\left(s,a_{i}\right)=\frac{\sum_{a_{j}\in A^{\prime }\left(s\right)}H(G|a_{j},s)}{|A^{\prime }\left(s\right)|} \end{array}$$

*where $a_{i}\in A\left(s\right),a_{j}\in A^{\prime }\left(s\right)$, and $A^{\prime }\left(s\right)=A\left(s\right)\backslash a_{i}$. The entropy $H(G|a_{j},s)$ computes the goal uncertainty contained in the reasoning result, and $H\left(G|a_{j},s\right)=-\sum p\left(G|a_{j},s\right)\cdot \log p\left(G|a_{j},s\right)$. Technically, we also need to remove zero entries of $p(G|a_{j},s)$, followed by normalization guaranteeing that sum(p)=1.*

The $rgu_{E}$ quantified by entropy *H* evaluates the goal uncertainty in a natural and compact way. More uncertainty introduced after the agent takes its action gives less information to goal recognizer and postpones the goal identification process. As the example in Figure 1 and assuming the agents are fully optimal, the $rgu_{E}$ for three actions leading to states (2,C), (1,B) and (1,D) are 0.32, 0.00 and 0.00 respectively. Also, the definition of $rgu_{E}$ could easily be applied to tasks where |G|>2. It should be noted that the definition of $rgu_{E}$ makes sure that this metric can be naturally applied to the situation where the agent changes its goal during the midway.

**Figure 4:**
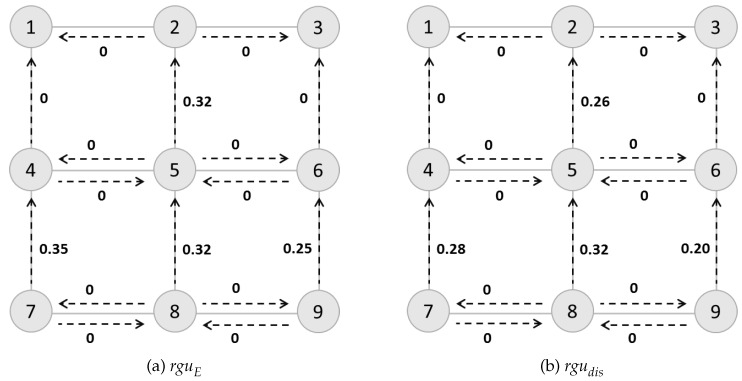
$rgu_{E}$ and the discounted $rgu_{dis}$ of each action in a 3×3 grid network

To illustrate how $rgu_{E}$ would be used in goal identification control problem and for clarity, we compute the values upon a 3×3 grid network (Figure 4a) with nodes representing grids and dashed edges connecting nodes. Selecting absorbing nodes No.1 and No.3 as possible goals, we compute $rgu_{E}$ for each action depicted as dashed line, as shown in Figure 4a. Higher $rgu_{E}$ means greater uncertainty associated with that action. Here we discuss two path-planning tasks *T* and $T^{\prime }$, where the agent has different starting points ($s_{0}=9$ or $s_{0}=8$). The tasks are defined as $T=\langle S,A,f,C^{\prime },G=\left\{1,3\right\},s_{0}\rangle$, with $C^{\prime }=C+rgu_{E}$ to model the road barrier, patrolling police or other methods the goal recognizer could use in order to control the goal identification process.

For the first task, the agent would choose h=〈9,8,7,4,1〉 for goal No.1 and h=〈9,6,3〉 for goal No.2. While for the second task, using the definition of $rgu_{E}$, the agent would choose one of the two optimal routes (h=〈8,7,4,1〉 or h=〈8,5,4,1〉), other than h=〈8,5,2,1〉 for goal No.1, and h=〈8,9,6,3〉 or h=〈8,5,6,3〉 other than h=〈8,5,2,3〉 for goal No.3. For the latter case, we find that $rgu_{E}$ cannot help the agent capture the fact in goal identification control that early interdiction would be more important than a later one.

To solve this problem, we define a discounted $rgu_{dis}$ so as to reduce the rgu value along with the increase of the number of steps that agent has taken from the start. For agent changing its goal during the midway, we could recompute this value from the original $rgu_{E}$. Case 3.1 shown in Section 4.1, which is talked about in the next section, gives a good indication of this situation occurrence.

**Definition** **3. (Discountedrgu *rgu*)** *The discounted rgu ($rgu_{dis}$) of a goal identification control problem is defined as:*

(13) $$rgu_{dis}\left(s,a_{i}\right)=rgu\left(s,a_{i}\right)\times \beta^{d}$$

*where β is the discount factor and d is the number of steps that agent has taken from the start.*

With the discount factor equals to 0.8, Figure 4b shows the discounted $rgu_{dis}$ values which have been adjusted according to its importance to the goal identification control task.

## 5. Goal Identification Control

In this section, we show how to control the rgu using the optimization technique. Instead of requiring the cost of cost-minimal plans to achieve each goal g∈G be the same before and after removing the subset of actions, as researched about in goal recognition design problem [6], we are more interested in deliberate changing goal uncertainty under adversarial settings where the resource constraint compared to optimality conservation is more applicable. Also, the original way of removing actions permanently is changed “softer” by adding additional costs to actions.

### 5.1. Reduction and Improvement of rgu

The goal identification control models based on optimization techniques are introduced. We first present models which are individually used for reducing and improving goal uncertainty for the different sides of the goal recognition task. Then we talk about the applicability of the offline-computed rgu in the online goal identification control.

The models could be transformed into the problem of maximizing (minimizing) the expectation of “s–t” path length, with the rgu being proportionally added to the original length. The mathematical-programming formulation of our new goal identification control model is as follows:

$$\begin{array}{ll} \mathrm{Problem}: & \mathrm{Maximize}\left(\mathrm{Minimize}\right)\mathrm{the expectation}“s-t”\;\mathrm{path length in a directed network by interdicting arcs},\\ \mathrm{Indices}: & i\in N,\mathrm{nodes in}\;G\;(s\;\mathrm{is the current source node},t\;\mathrm{is the terminus}),\\ & k=(i,j)\in A,\mathrm{arcs in}\;G,\\ & k\in FS(i)(k\in RS(i)),\mathrm{arcs directed out of}\;(\mathrm{into})\mathrm{node}i,\\ \mathrm{Data}: & c_{k}=1,\mathrm{normal length of arc}\;k\;\left(\mathrm{vector form}\;c\right),\\ & d_{k}=1,\mathrm{added integer delay if arc}\;k\;\mathrm{is interdicted}\;\left(\mathrm{vector form}\;d\right),\\ & rgu_{k},\mathrm{relative goal uncertainty of arc}\;k\;\left(\mathrm{vector form}\;\mathrm{rgu}\right)\\ & r_{k}=1,\mathrm{resource required to interdict arc}\;k\;\left(\mathrm{vector form}\;r\right),\\ & R,\mathrm{total amount of interdiction resource},\\ \mathrm{Variables}: & x_{k}=1\;\mathrm{if arc}\;k\;\mathrm{is interdicted by the interdictor};\mathrm{else}x_{k}=0,\\ & y_{k}=1\;\mathrm{if arc}\;k\;\mathrm{is traversed by the evader};\mathrm{else}y_{k}=0 \end{array}$$

The formulation of *rgu* reduction problem (**[RGUR-P]**) for the observer is:

$$\left[\mathrm{RGUR}-P\right]\;\max_{x\in X}\sum_{k\in A}\left(c_{k}+x_{k}d_{k}\left(1+rgu_{k}\right)\right)y_{k}$$

(14) $$\sum_{k\in FS(i)}\;\;\;y_{k}\;-\;\;\;\sum_{k\in RS(i)}\;\;\;y_{k}\;\;=\;\;\left\{\;\;\begin{array}{cc} 1 & \;\mathrm{for}\;i=s\\ 0 & \;\forall i\in N\backslash \{s,t\}\\ -1 & \;\forall i=t \end{array}\right.$$

(15) $$x_{k},y_{k}\in \left\{0,1\right\},\;\forall k\in A$$

where $X=\{x\in {\left\{0,1\right\}}^{\left|A\right|}|r^{T}x\leq R\}$, Equation (14) is the flow-balance constraint.

While the formulation of *rgu* improvement problem (**[RGUI-P]**) for the observed agent is:

$$\left[\mathrm{RGUI}-P\right]\;\min_{y}\sum_{k\in A}\frac{\left(c_{k}+x_{k}d_{k}\right)y_{k}}{1+rgu_{k}}$$

(16) $$\sum_{k\in FS(i)}\;\;\;y_{k}\;-\;\;\;\sum_{k\in RS(i)}\;\;\;y_{k}\;\;=\;\;\left\{\;\;\begin{array}{cc} 1 & \;\mathrm{for}\;i=s\\ 0 & \;\forall i\in N\backslash \{s,t\}\\ -1 & \;\forall i=t \end{array}\right.$$

(17) $$x_{k},y_{k}\in \left\{0,1\right\},\;\forall k\in A$$

where $X=\{x\in {\left\{0,1\right\}}^{\left|A\right|}|r^{T}x\leq R\}$. Thus, with additional goal uncertainty information, the problem could be transformed into network interdiction models, which are of a typical mixed-integer program (MIP) and solved using linear programming algorithms.

**Figure 5:**
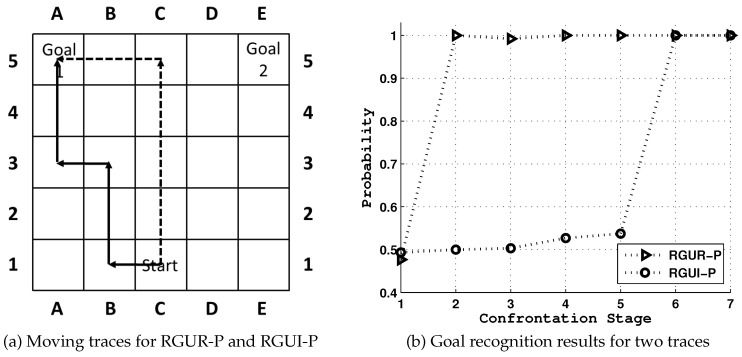
The moving traces (the solid line for agents moving after the observer’s interdiction; dashed line for the observed agent selecting the most ambiguous path) and their corresponding recognition results p(G|O) for the example shown in Figure 1.

Figure 5 shows two moving traces of the observed agent under [RGUR-P] and [RGUI-P] problems. The observer could use [RGUR-P] to reduce the enemy’s goal uncertainty and accelerate situation awareness, while the observed agent, after analyzing the observer’s goal recognition task, could actually use [RGUI-P] to cover its real intention to the maximum extent. General results would be given in Section 6.

Please note that until now, the metric rgu used for assessing goal uncertainty is defined over actions available at each state in an offline manner, i.e., $p(G|a_{i},s)$ are computed according to P(G|O)=αP(O|G)P(G), with prior probability P(G) following Uniform distribution, instead of over agent’s full course of actions where the real-time $P^{\prime }\left(G\right)$ contains history information, this may incur uncertainty inconsistency among individual states and the whole task. Fortunately, our definition of *rgu* has no effect on problem-solving of the goal identification control,

Case 1: If the real-time prior $P^{\prime }\left(G\right)$ cannot help observer distinguish the right goal, meaning that $H(P^{\prime }\left(G\right))$ is relatively high. Then it is naturally established for agents choosing actions with either high or low uncertainty.

Case 2: If $P^{\prime }\left(G\right)$ distinguishes the goals clearly where $H(P^{\prime }\left(G\right))$ is relatively low, and agent chooses the action that has high uncertainty, i.e., rgu≠0, then highly uncertain action would input little information to the original beliefs about agent goals. The agent’s belief of the right goal would still maintain. However, in this case, resource needed for action interdiction would be wasted as little information would be given and agent’s intention usually maintains for a period.

Case 3: Also, if $P^{\prime }\left(G\right)$ distinguishes the goals clearly, e.g., the prior $P^{\prime }\left(g\right)$ for goal *g* with the largest value, while agent chooses the action that has low uncertainty *rgu*, then two cases should be considered. Firstly, agent’s belief of the right goal has changed according to the posterior P(G|o) (Case 3.1). This happens when the agent is changing its goal from *g* to $g^{\prime }$ during the midway, and thus chooses the action that will lead to $g^{\prime }$. Secondly, the computed posterior P(G|o) still capture the right goal *g* (Case 3.2). For both cases, the *rgu* works properly.

### 5.2. The SPLUNI Model and Its Dual Form

After individually talk about *rgu* reduction and improvement, in this section we propose a SPLUNI model, where uncertainty becomes another optimization objective along with path length. The SPLUNI model could be viewed as a bilevel mixed-integer program (BLMIP).

The problem description of SPLUNI is similar to [RGUR-P] and [RGUI-P], whereas it is described as maximizing the expectation of the shortest s−t path length while minimizing the largest uncertainty and $0\leq c_{k},d_{k},r_{k}<\infty$. Its mathematical-programming formulation is given as:

$$\left[\mathrm{SPLUNI}-P\right]\;\max_{x\in X}\min_{y}\sum_{k\in A}\frac{\left(c_{k}+x_{k}d_{k}\left(1+\alpha \times rgu_{k}\right)\right)y_{k}}{1+\beta \times rgu_{k}}$$

with the same constraints as Equations (14) and (15) and $r^{T}x\leq R$. α,β are parameters controlling the importance of goal uncertainty between two objectives.

As a BLMIP problem, which cannot be solved directly using the MIP approaches, we propose a dual reformulation of SPLUNI. Fix the outer variable *x* and take the dual of the inner minimization using KKT conditions, the final MIP formulation is given as:

$$\left[\mathrm{SPLUNI}-D\right]\;\max_{x\in X,\vec{\pi }}b^{T}\vec{\pi }$$

(18) $$s.t.\;K^{T}\vec{\pi }=c^{\prime }+D^{\prime }x$$

(19) $$\pi_{s}=0$$

where K is the network matrix controlling y as in Equation (14), $r^{T}x\leq R$, $b={\left(1,0,\cdots ,0,-1\right)}^{T}$, $c_{k}^{\prime }=c_{k}/\left(1+\beta \times rgu_{k}\right)$ and $d_{kk}^{\prime }=d_{kk}\times \left(1+\alpha \times rgu_{k}\right)/\left(1+\beta \times rgu_{k}\right)$.

## 6. Experiments

For simplicity, we select a reduced Chicago Sketch Road Network [20] expanded from the vertex No.368 to its neighbors and neighbors’ neighbors for 5 times, consisting of 51 vertexes and 113 edges. The computations of the RGUR and RGUI are formulated into a BLMIP, and SPLUNI into a BLMIP and solved using the MIP solvers of CPLEX 11.5 and YALMIP toolbox of MATLAB [57]. Nontrivial details are omitted.

### 6.1. Tests on Uncertainty Reduction

**Figure 6:**
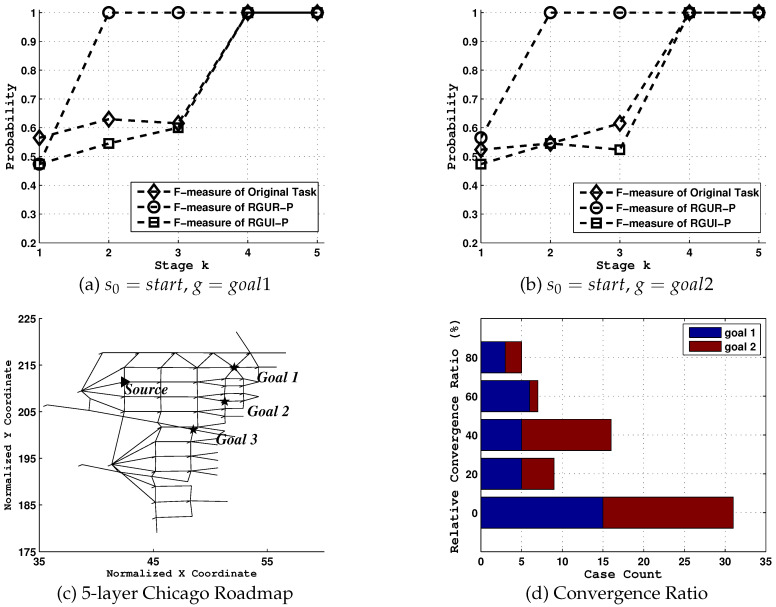
The statistical evaluation of uncertainty influence on goal recognition upon a reduced sketch road network.

Upon the reduced Chicago Sketch Road Network, a goal recognition task is defined where $s_{0}=start$, G={goal1,goal2} as shown in Figure 6c. We first show the influence of uncertainty on the goal recognition system and how *rgu* reduction model could be used in advancing situation awareness.

In this test, a dataset consisting of 100 labeled traces for each goal is collected using agent decision model, and we use *F-measure* which is a frequently used metric to measure the overall accuracy of the recognizer [1], computed as:

(20) $$F-\mathrm{measure}=\frac{2\cdot \mathrm{precision}\cdot \mathrm{recall}}{\mathrm{precision}+\mathrm{recall}}$$

(21) $$\mathrm{precision}=\frac{1}{N}\sum_{i=1}^{N}\frac{{\mathrm{TP}}_{i}}{{\mathrm{TI}}_{i}}$$

(22) $$\mathrm{recall}=\frac{1}{N}\sum_{i=1}^{N}\frac{{\mathrm{TP}}_{i}}{{\mathrm{TT}}_{i}}$$

where *N* is the number of possible goals, ${\mathrm{TP}}_{i}$, ${\mathrm{TI}}_{i}$ and ${\mathrm{TT}}_{i}$ are the true positives, total of true labels, and the total of inferred labels for class *i* respectively. Equations (20) to (22) show that *F-measure* is an integration of precision and recall, where *precision* is used to scale the reliability of the recognized results and *recall* to scale the efficiency of the algorithm applied in the test dataset. The value of *F-measure* will be between 0 and 1, and a higher metric value means a better performance. As to evaluate traces with different lengths, the paper applies the method in [35], and partitions each trace into *k* $\left(k=1,2,...,N_{\mathrm{stage}}\right)$ stages. The corresponding sequences are $y_{t\in i:\lceil k*{\mathrm{length}}^{j}/N_{\mathrm{stage}}\rceil }^{j\in 1:N_{\mathrm{trace}}}$.

From the results shown in Figure 6a,b, goal uncertainty associated with certain domains indeed seriously impedes effective goal identification. Under [RGUR-P] model, the values of *F*-measure after the network redesignation increases to almost 1 the moment agent selects its first action, compared to values under tasks with no network changes and the most ambiguous situation ([RGUI-P]). While for the RGUI-P model, its *F-measure* values are equal or greater than those of the original task in most times. Note the big difference of *F-measure* values between two models, i.e., [RGUR-P] and [RGUI-P], this simple case not only proves that the goal uncertainty could be purposefully controlled in both directions, but also shows the potential advantage that the observer could take off during the goal identification process.

Next, we statistically evaluate the maximum early prediction that our model enables according to *Convergence Point* metric [1], defined as 0<τ=CPoint≤T, s.t.$p_{\tau }\left(g_{true}|o_{\tau }\right)\geq \gamma$. CPoint denote the time step at which the posterior of the agent’s true goal is greater or equal to β. For tests in Figure 6d, γ is set to 0.8. We test recognition tasks $D=\{s_{0},G=\left\{goal1,goal2\right\}\}$ for $\forall s_{0}\in S\backslash \left\{G,U\right\}$, where *U* is the set of nodes with no available paths to *G*, and compare the early convergence ability of goal identification between [RGUR-P] and [RGUI-P] using a new metric RelConvergeRatio, which describes the relative convergence ratio (rcr(%)) between two models given the $s_{0}-g_{true}$ path length,

(23) $$RelConvergeRatio=\frac{CPoint_{I}-CPoint_{R}}{|h=\langle s_{0},\ldots ,g_{true}\rangle |-1}$$

where $\left|h=\langle s_{0},\ldots ,g_{true}\rangle \right|$ is the length of path starting from $s_{0}$ while ending at the real goal $g_{true}$.

Clearly, both for tasks where $g_{true}$ equals to goal1 and goal2, there are considerable reductions of goal uncertainty. For example, there are total 5 cases in these two tasks whose relative convergence ratio is greater than or equal to 80%. In addition, 7 cases in total fall within the 60%–80% interval, 16 at 40%–60% and 9 at 20%–40%. It should also be noted that there are more than 30 cases whose *RelConvergeRatio* equals to 0. This means that although the goal uncertainty widely exists in many identification tasks, it does not exist in every case.

Further for generality, we test 3 more cases where $G_{1}=\left\{goal2,goal3\right\}$, $G_{2}=\left\{goal1,goal3\right\}$ and $G_{3}=\left\{goal1,goal2,goal3\right\}$, as shown in Table 1. From these results, we could conclude that:

- goal uncertainty exists in normal goal recognition task, and it could be purposefully controlled to affect the agent’s situation awareness.
- the possible reduction of goal uncertainty differs in each scenario.

### 6.2. Performance of SPLUNI

Finally, we test the performance of SPLUNI (set α=β=1), which is the game between the observer (interdictor) and the observed agent (evader), in maximizing the expectation of the shortest s−t path length while minimizing the largest uncertainty, as shown in Table 2. We first define the path interdiction efficiency as $e=\Delta L/(d^{T}\times x)$ where ΔL is the increased path length for evader after interdiction and $d^{T}\times x$ is the total length increase in the network. The tests are conducted over those ambiguous cases in three goal settings. E(e) and E(rcr) help us understand the impact of SPLUNI model on both network interdiction and goal uncertainty reduction.

Clearly, SPLUNI model works well for observer by adding the evader’s path length and at the same time reducing the goal uncertainty during the process. In all three goal settings, the average path interdiction efficiencies are more than 50%. Especially when G={g2,g3}, E(e) reaches to almost 80% for $g_{true}=g2$ and more than 90% for $g_{true}=g3$. This proves the effectiveness of SPLUNI model in network interdiction. Also, the model successfully reduces the goal uncertainty to a certain level in three different settings, as we could find in the data of E(rcr). The above results show that the SPLUNI model that we propose is of great value for security domains where high-value targets need to be timely recognized meanwhile the intruder’s actions be delayed.

## 7. Conclusions

We present a new perspective of goal identification control using off-the-shelf probabilistic goal recognizer, and introduce the *relative goal uncertainty* (*rgu*) value for actions in a goal recognition task. We present ways of controlling goal uncertainty, followed by a presentation of the method in an adversarial setting using a SPLUNI model. Empirical evaluation shows the effectiveness of the proposed solution in controlling goal uncertainty in recognition tasks.

The work grounds the behaviors of the agents on clear, well-founded computational models. Inference over graphic models and mixed-integer programming is brought together to provide a compelling solution to the goal identification problem. Though this work is quite suggestive but still simple, which means there are many open problems in our future work. Currently, as there exists inconsistency of goal uncertainty between the offline P(G) which follows Uniform distribution and the real-time P(G|O) assessed online according to the agent’s observations, how to incorporate the goal uncertainty information into the goal identification control process online has not yet been solved. Also, we will also try to incorporate rgu definition into the plan identification process to improve the method’s applicability.

## Figures and Tables

**Table 1:** The number of cases (discard states unreachable to $g_{true}$) fall in intervals of relative convergence ratio (rcr(%)). “/” separates results with elements in *G* being the true goal.

G\rcr(%)	0	≤20	≤40	≤60	≤80
{goal2,goal3}	23/24	1/3	3/3	2/0	1/0
{goal1,goal3}	25/31	2/4	6/0	0/0	0/0
{g1,g2,g3}	15/21/19	3/6/1	4/1/4	3/1/1	0/0/0

**Table 2:** The expectations of path interdiction efficiency and relative convergence ratio (rcr(%)) for different goal setting.

	{g1,g2}	{g1,g3}	{g2,g3}
E(e)	56.0/73.6	63.4/67.0	77.4/90.7
E(rcr)	19.7/13.3	7.4/9.1	2.3/1.9

## Abbreviations

The following abbreviations are used in this manuscript:

AHMM	Abstract Hidden Markov Models
DBN	Dynamic Bayesian Network
GRD	Goal Recognition Design
PRD	Plan Recognition Design
MDP	Markov Decision Process
MIP	Mixed-Integer Programming
rgu	relative goal uncertainty
KKT	Karush-Kuhn-Tucker condition
wcd	worst-case distinctiveness
SPLUNI	Shortest-Path Largest-Uncertainty Network Interdiction
RGUR	rgu Reduction
RGUI	rgu Improvement
